# Bullet-induced synovitis as a cause of secondary osteoarthritis of the hip joint: A case report and review of literature

**DOI:** 10.1186/1752-1947-1-171

**Published:** 2007-12-05

**Authors:** Muhammad A Rehman, Masood Umer, Yasir J Sepah, Muhammad A Wajid

**Affiliations:** 1 Resident Section of Orthopedics, Department of Surgery Aga Khan University Hospital, Karachi-74800, Pakistan; 2 Assistant Professor Section of Orthopedics, Department of Surgery Aga Khan University Hospital, Karachi-74800, Pakistan; 3 Department of Surgery (Orthopedics) Aga Khan University Medical College, Karachi-74800, Pakistan

## Abstract

**Background:**

With increasing prevalence of gunshot injuries we are seeing more patients with retained bullet fragments lodged in their bodies. Embedded lead bullets are usually considered inert after their kinetic energy has dissipated hence these are not removed routinely. However, exposure of any foreign body to synovial fluid may lead to rapid degradation and hence result in systemic absorption, causing local and systemic symptoms. We present the case of a thirty year old man who came to our out patient department with a history of progressive, severe hip pain ten years after a gun shot injury to his right hip.

**Conclusion:**

The common belief that intraarticular bullets should not be removed has no benefit and may result in unwanted long term complications.

## Introduction

With increasing prevalence of gunshot injuries we are seeing more and more patients with retained bullet fragments lodged in their bodies [1]. Embedded lead particles are usually considered inert after their kinetic energy has dissipated hence these are not removed routinely. Removal is indicated if they impinge on vital structures or are easily accessible during operation for other reasons [2-5]. A review of literature shows that retained intra-articular bullets have been associated with significant morbidity [6-9], joint degeneration and ultimately resulting in joint replacement.

Intra-articular bullet fragments behave differently due to direct contact with synovial fluid. Lead being soluble in synovial fluid [3,10] can cause both local and systemic effects. Lead poisoning from retained intra articular bullets has been recognized in the literature since 1867 [3,11-13]. Although in most of the cases the cause of arthropathy is not known but it's attributed mainly to mechanical forces along with local effects of lead poisoning [3,4,6,7,10,14]. A retained bullet can not only produce foreign body reaction, mechanical articular cartilage damage and proliferative synovitis, leading to destructive arthritis but can also lead to systemic absorption of lead. However, there is considerable variation in extent of lead absorption, onset of time to symptoms, severity of symptoms and toxicity [13]. Symptoms of systemic lead poisoning are usually vague; headache, nausea, fatigue and abdominal pain [15].

Radiographic identification of intra-articular bullet fragments should prompt an urgent orthopedic consultation [9] as timely removal can prevent both lead arthropathy and systemic toxicity [10].

## Case Presentation

**Figure 1:**
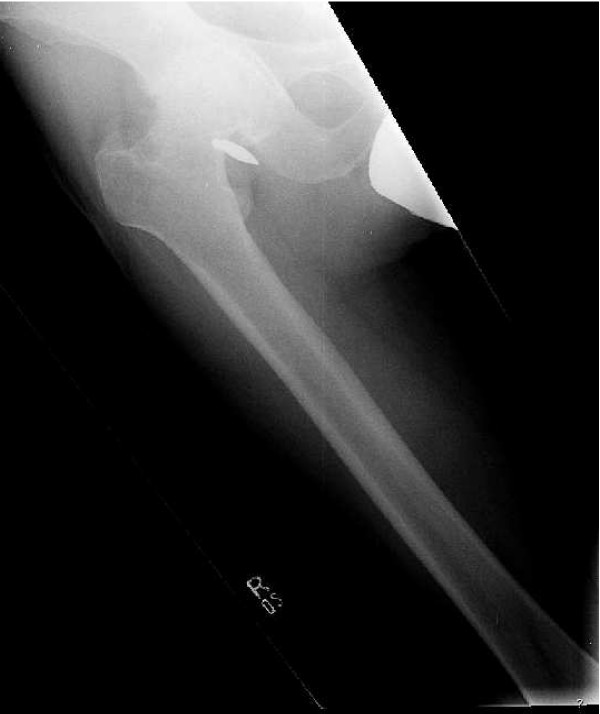
Showing presence of intraarticular bullet in right hip joint and arthritis.

**Figure 2:**
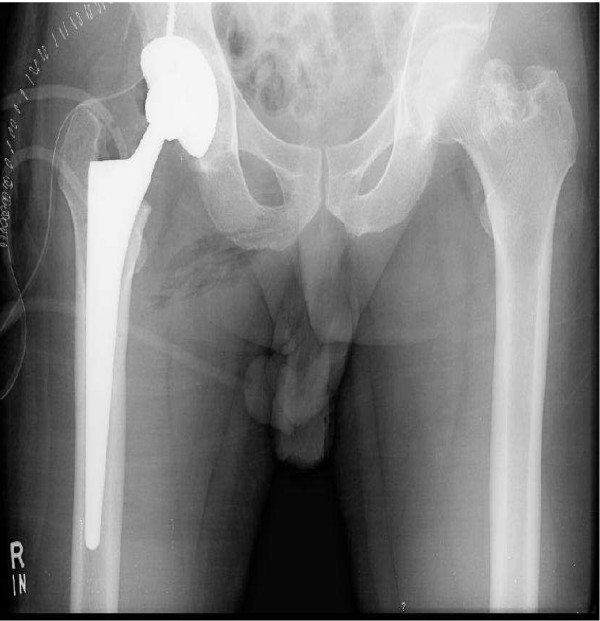
Showing postoperative radiograph after total hip arthroplasty.

**Figure 3:**
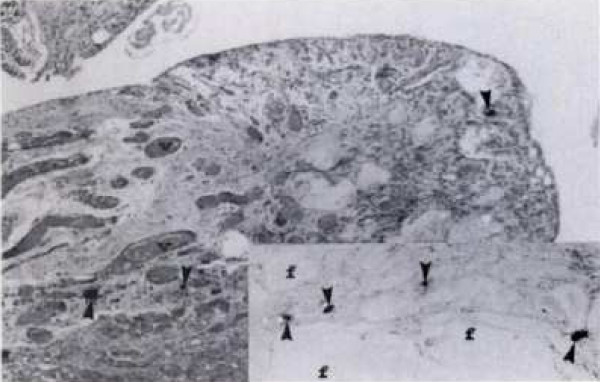
Arrows showing lead deposits in synovium.

Thirty eight years old male presented with a history of progressive, severe hip pain ten years after a gun shot injury to his right hip. Radiographs at the time of injury confirmed the presence of bullet around the hip joint. He was managed conservatively at that time. Now he was complaining of hip pain for the last two years which had progressively increased significantly over the last six months. Clinically the patient had limited and painful range of motion with 20 degrees of fixed flexion contracture. Current radiographs revealed a bullet fragment inside the hip joint with severe degenerative arthritis (figure 1). Considering the intractable pain and advanced arthritis a right total hip arthroplasty was done. At the time of surgery, about fifty milliliters of fluid was removed from the joint and sent for culture and sensitivity, which turned out to be negative for any microorganism. There was extensive synovitis inside the degenerated acetabulum. The loose bullet fragment was removed easily and an un-cemented total hip arthroplasty (Protek, Mathys Medical) was performed (figure 2). Lead deposits were seen in the synovium (figure 3). Postoperative course was uneventful and at eight months follow up the patient could bear full weight on his right leg.

## Discussion

Rapid encapsulation of most foreign bodies composed of lead occurs via fibrosis, and this process essentially removes them from exposure to circulating body fluid with a subsequent drop in serum lead levels [13,16,17]. However, exposure of a leaded bullet to synovial fluid leads to rapid degradation and hence result in systemic absorption, causing local and systemic symptoms of lead intoxication [3,4,6,7,10,14]. Two factors responsible for the dissolution of lead fragments in synovial fluid are the presence of hyaluronic acid and the ph of synovial fluid [8]. On the other hand mechanical destruction of joint may be caused by several factors. Firstly the initial trauma may cause fractures of articular bone, leading to an incongruous and irregular joint surface. Motion of such surfaces against each other may lead to joint destruction. Secondly, when a bullet hits the bone; its articular cartilage, bone and pieces of lead may fragment, leading to intra articular debris that can pit and erode the joint surfaces. Thirdly, a bullet embedded in bone may extend partially into the joint; further motion can results in additional destruction of cartilage [10-12,14,18]. Toxic histologic manifestations of intra-articular lead have also been reported in animal models by Bolanos et al [19] and Harding et al [20]. Harding et al [20] studied the effects of intra-articular lead implants on the synovium, articular cartilage and meniscus of white rabbits at 4, 6, 10 and 14 weeks. Articular and meniscal changes that Harding et al came across were chondrocyte proliferation, disorganization of the columnar epithelium. Tide mark duplication and unequal thickness of the cartilage was observed in the articular cartilage while the synovium showed both cellular and stromal hyperplasia [20].

If lead arthropathy is identified, removal of lead fragments [14,15,21] is mandatory along with other procedure/s as indicated by the condition of the joint. Intraarticular lead poisoning has been reported in the literature in the context of gout, synovitis and degenerative joint diseases along with systemic lead poisoning [3,4,7,10,13,14,16,22,23]. All patients with lead arthropathy should be evaluated for systemic lead toxicity [18].

## Conclusion

Although bullet dislodgement into the joint space is very rare, its urgent removal is warranted if found. Its early removal will prevent both local and systemic lead intoxication. If not removed, it can result in lead arthropathy ultimately resulting in joint replacement. The common belief that intra-articular bullets should not be removed has no benefit and might cause a lot of long term complications.

## Competing interests

The author(s) declare that they have no competing interests.

## Authors' contributions

MAR conceived of the case, drafted the manuscript and did the literature review. MU helped in drafting and reviewed the case. MAW reviewed the case, helped in drafting the report. YJS helped in literature review and formatting the material. All authors read and approved the final manuscript.

## Consent

The authors confirm that a formal written consent was taken for the publication of this case report.
